# The use of medical health applications by primary care physicians in Israel: a cross-sectional study

**DOI:** 10.1186/s12913-024-10880-w

**Published:** 2024-04-02

**Authors:** Menashe Meni Amran, Avital Bilitzky, Mattan Bar-Yishay, Limor Adler

**Affiliations:** 1 Ben-Gurion University of the Negev, Beer-Sheva, Israel; 2 Department of Family Medicine, Maccabi Healthcare Services, Tel Aviv, Israel; 3 Department of Family Medicine, Sackler Faculty of Medicine, Tel Aviv University, Tel Aviv, Israel

**Keywords:** Medical health applications, Primary care physicians, Attitudes

## Abstract

**Background:**

The use of medical health applications (mHealth apps) by patients, caregivers, and physicians is widespread. mHealth apps are often employed by physicians to quickly access professional knowledge, guide treatment, easily retrieve medical records, and monitor and manage patients. This study sought to characterize the use of mHealth apps among primary care physicians (PCPs) in Israel. The reasons for using apps and barriers to their use were also investigated.

**Methods:**

From all MHS’ PCPs, we randomly selected 700 PCPs and invited them to complete a questionnaire regarding the use of mHealth apps and attitudes toward them.

**Results:**

From August 2020 to December 2020, 191 physicians completed the questionnaire (response rate 27.3%). 68.0% of PCPs reported using mHealth apps. Telemedicine service apps were the most frequently used. Medical calculators (used for clinical scoring) and differential diagnosis apps were the least frequently used. The most common reason for mHealth app use was accessibility, followed by time saved and a sense of information reliability. Among infrequent users of apps, the most common barriers reported were unfamiliarity with relevant apps and preference for using a computer. Concerns regarding information reliability were rarely reported by PCPs. Physician gender and seniority were not related to mHealth app use. Physician age was related to the use of mHealth apps.

**Conclusions:**

mHealth apps are widely used by PCPs in this study, regardless of physician gender or seniority. Information from mHealth apps is considered reliable by PCPs. The main barrier to app use is unfamiliarity with relevant apps and preference for computer use.

**Supplementary Information:**

The online version contains supplementary material available at 10.1186/s12913-024-10880-w.

## Introduction

Medical health applications (mHealth apps) are defined by the United States (US) Food and Drug Administration (FDA) as software functions deployed on a mobile platform [1]. Most mHealth apps are aimed at public health. These include apps that focus on exercise, weight loss, diabetes management, and women’s health [2]. Some mHealth apps are designated for health professionals, including medical calculators, informative apps, and organizational apps (designed for working purposes).

The use of medical health applications (mHealth apps) by patients, caregivers, and physicians is widespread [3–6]. The use of these apps among physicians rose from 68% in 2012 to 84% in 2015 [7]. Apps are often employed by physicians to quickly access professional knowledge, guide treatment, train students and residents, easily retrieve medical records, consult specialists, and monitor and manage patients [8–12]. Georgetown University’s medical school now requires its students to own and use personal smartphones from the start of the program, highlighting how smartphones have become an integral part of medical practice [13].

A British survey revealed that most physicians and medical students use one to five mHealth apps, while a minority regularly uses more than ten [14]. The use of mHealth apps is also prevalent in primary care [15–17], where even instant messaging apps such as WhatsApp are widely employed as convenient and accessible telemedicine consultation tools [18–20].

While mHealth apps can be used to manage chronic diseases and increase adherence to drug therapy, evidence for their effectiveness in these realms is not clear [21]. The adoption of these apps by physicians and healthcare professionals is influenced by social, organizational, and technological factors [6, 22].

The widespread use of mHealth apps, instant messaging consultations, and even social media raises questions regarding the need for ethical and professional boundaries in clinical practice [23]. It appears that the lack of standardization and professional validation of mHealth apps is a major barrier to professionally acceptable use [24, 25]. In 2019, the FDA announced that it would begin regulating medical applications used to diagnose medical conditions or guide or suggest medical treatment [26].

Previous studies have described the use of mHealth apps among primary care physicians (PCPs) in Turkey, Sweden, China, Germany, France, Australia, Austria, and Belgium [27–35]. To the best of our knowledge, the use of mHealth apps among PCPs in Israel had not yet been studied prior to our study. The aim of this study was to investigate the use of mHealth apps among PCPs in Israel, including the barriers to use, determine the frequency of their use, and reveal whether various personal characteristics of PCPs are associated with frequent app use.

## Methods

### Setting and study design

This cross-sectional study was conducted among PCPs employed by Maccabi Healthcare Services (MHS), the second-largest health maintenance organization (HMO) in Israel, which covers more than 2.6 million patients nationwide. From August 2020 to December 2020, we sent an online questionnaire via organizational e-mails to 700 MHS PCPs and asked them about their usage of mHealth apps. Reminders were sent three times. The study was approved by the MHS institutional review board (0014-1-BBL).

### Participants

Around 1,300 PCPs work in MHS (for salary or per-fee service). We randomly selected 700 PCPs and sent them the survey. The inclusion criteria were working as a PCP in MHS (including residents). There were no exclusion criteria.

### The questionnaire

The lead author designed the questionnaire based on similar previous studies [27, 32, 33, 36]. All other authors reviewed and revised the questionnaire, and it was later validated by a group of ten PCPs. The questionnaire consisted of several sections. In the first section, respondents were asked whether they owned a smartphone and were questioned regarding their use of apps in general and mHealth apps in particular, including frequency of use. In the second section, respondents were asked about specific types of mHealth apps (medications and dosages, medical calculators, access to information, differential diagnosis, treatment options, telemedicine), the reasons for using mHealth apps, factors that would encourage them to use these apps more often, and barriers to their use of these apps. In the third section, respondents were asked whether they recommended mHealth apps to their patients, whether they were interested in participating in educational programs regarding mHealth apps, and what they thought about their future use of mHealth apps. In the fourth and last section of the questionnaire, respondents were asked about their demographics and personal experience, including age, gender, years of experience working in the community, residency status (resident/specialist), whether their practice was urban or rural, and additional information.

The questionnaires were anonymous, and no identifying information was collected. Consent to participate was granted by submission of a completed questionnaire. The questionnaire was administered in Hebrew. An English translation of the questionnaire was done by the lead researcher (available as supplementary material 1).

### Statistical analysis

Descriptive data were reported as mean and standard deviation (SD) for continuous variables and percentages for categorical variables. The effects of noted barriers or contributing factors on mHealth app use were assessed using the Chi-square test for categorical variables and the t-test for continuous variables. All p-values were two-sided and statistical significance was set at *P* ≤ 0.05. Statistical analysis was performed using IBM SPSS version 25.

## Results

### Participants

The questionnaire was answered by 191 PCPs (response rate 27.3%). Of these, 10 physicians reported not using smartphones and, therefore, were not included in the analysis; 133 (69.6%) were PCP specialists, and 14 (7.3%) were residents. The mean age was 53.3 ± 12.7 (median 55, IQR = 41–65) (Table 1).

**Table 1:** Characteristics of the respondent physicians (*N* = 191)

Characteristics	Respondent physicians
Gender, % (n)	
Men	45.5% (87)
Women	54.5% (104)
Age, mean ± SD	53.3 ± 12.7
Country of birth, % (n)	
Israel	41.4% (79)
Elsewhere Missing	39.7% (76) 18.8% (36)
University Studies (medical degree), % (n)	
Israel	42.9% (82)
Elsewhere Missing Years working as a physician in the community, mean ± SD (years)	36.7% (70) 20.4% (39) 25.4 ± 13.7
Workplace (*), % (n)	
Urban primary care clinic	65.4% (125)
Rural primary care clinic	8.4% (16)
Public hospital	4.2% (8)
Elsewhere Missing	2.6% (5) 19.4% (37)
Specialization (**), % (n)	
General practitioner without specialization, %	23.0% (44)
Family medicine resident	7.3% (14)
Family medicine specialist	45.5% (87)
Internal medicine specialist	24.1% (46)
Smartphone user, % (n)	
Yes No	94.8% (181) 5.2% (10)
mHealth App Users among Smartphone Users	
Yes No	68.0%(123) 32.0% (58)

(*) The workplace (urban/rural) was subjectively defined by the respondents

(**) In Israel, there is a 4-year residency program in primary care. Afterwhich, the physician is considered a specialist in Family Medicine

### Descriptive data

**Fig. 1:**
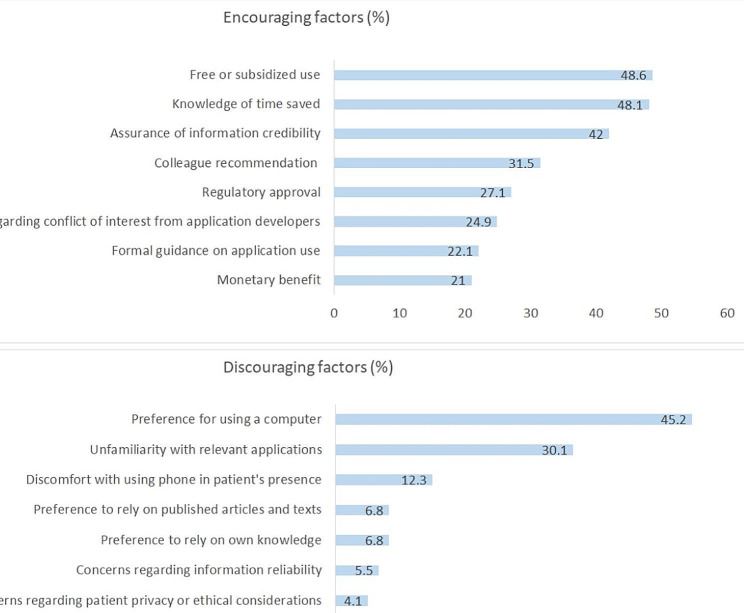
Factors that encourage or discourage medical health application use

One hundred and twenty-three (123) PCPs (68%) reported using mHealth apps (available as supplementary material 2). When asked about mHealth apps use frequency, 21/181 (11.6%) reported using them several times a day, 25/181 (13.8%) at least once daily, 62/181 (34.3%) several times weekly, 33/181 (18.2%) at least once that week, and 40/181 (22.1%) reported not using them at all. Meaning, most respondents use them on a regular basis in practice. Telemedicine service apps were the most frequently used mHealth apps. Forty-one (41) PCPs (23%) reported using telemedicine apps (including the MHS app and WhatsApp) several times a day. The least frequently used apps were medical calculators (used for clinical scoring) and differential diagnosis applications, which 93 physicians (51%) reported never using. The most common reasons cited for use were accessibility (reported as the main reason for mHealth app use by 97 physicians [54%]), time efficiency (92 physicians, 51%), and a sense that the information is up-to-date and reliable (92 physicians, 51%). Time efficiency was also reported by 87 physicians (48%) as a factor that would encourage app use, alongside subsidized or discounted fees (88 physicians, 49%) and assurances of information credibility (76 physicians, 42%) (Fig. 1).

Among infrequent users of medical applications (*n* = 73), the most commonly reported barriers were preferring a computer rather than a phone (reported by 33 physicians [45%]) and not being familiar with relevant applications (reported by 22 physicians [30%]). Concerns regarding information reliability, patient privacy, or other ethical implications were rarely reported by physicians as barriers (reported by 5% or less of physicians) (Fig. 1).

One hundred and three (103) physicians (57%) reported that they believed their use of mHealth apps would increase in the future. Thirty-eight (38) physicians (21%) noted interest in participating in formal training on mHealth apps, and 59 physicians (33%) responded they would probably like to participate in such training. However, only 40 physicians (22%) declared that formal training would encourage them to increase their use of mHealth apps. Indicating an acceptance of the phenomenon and willingness to embrace its potential developments in an informed manner. Thirty eight (38) physicians (21%) declared that monetary benefit would encourage them to increase their use of medical applications. Senior physicians were more likely than young physicians to indicate discounted or subsidized fees as an encouraging factor for application use (*p* = 0.01).

Notably, while most physicians use mHealth apps, only a small number recommend their use to patients. Only 11 (6.1%) physicians in our study reported frequently recommending applications to patients.

Physician gender, country of birth and seniority, were not related to mHealth app use. Physician age was related to app use (*p* = 0.03); app users (mean age 51.5) were younger than non-users (mean age 55.9). However, frequency of mHealth app use among reported users was not related to physician age. Meaning while app users are on average younger, how often apps are used was not correlated with age within this group. Medical residents were also more likely than other physicians to use mHealth apps (*p* = 0.03); 93% of residents reported using them, compared to only 55%, 66%, and 75% of physicians with no specialty, family medicine specialists, and internal medicine specialists, respectively (Table 2). This difference was due to the use of apps for drug dosages or clinical scoring, which was frequent among residents but rare among other physicians. Indicating that while the more common uses for mHealth apps, such as telemedicine, are common in the practice of all physicians, less experienced physicians use digital tools to enhance and improve their developing clinical practice.

**Table 2:** Factors related to medical health application use

Characteristics	Physicians who use mHealth apps (*N* = 123)	Physicians who do not use mHealth apps (*N* = 58)	PV
Gender, % (n)			0.250
Men	48.8% (60)	39.7% (23)	
Women	51.2% (63)	60.3% (35)	
Age, mean ± SD	51.5 ± 12.7	55.9 ± 12.5	0.03
Country of birth, % (n)			
Israel	48.0% (59)	31.0%(18)	
Elsewhere Missing	35.8% (44) 16.3% (20)	41.4% (24) 27.6% (16)	0.063
Seniority, mean ± SD (years)	23.8 ± 13.9	26.8 ± 12.6	0.240
Specialization, % (n)			
General practitioner without specialization, %	17.1% (21)	29.3% (17)	0.047
Family medicine resident	10.6% (13)	1.7% (1)	
Family medicine specialist	45.5% (56)	50.0% (29)	
Internal medicine specialist	26.8% (33)	19.0% (11)	

*Chi-square was used to compare categorical variables and Independent T-test was used to compare continuous variables

## Discussion

### Principal results

This cross-sectional study explored the use of mHealth apps among PCPs in Israel. The main findings include a high reported rate of use of mHealth apps, with telemedicine apps the most common (including the MHS app and WhatsApp). Medical calculators were the least common app type used. Reasons for the use of mHealth apps were their accessibility and PCPs’ sense that they save time and are up-to-date and reliable. Barriers to usage were the preference for computers over phones and lack of familiarity with these apps. Most PCPs believe they will use mHealth apps more in the future and are interested in receiving training in this field.

### Limitations

The limitations of this study are its relatively low response rate (27.3%) and the fact that it covered only one HMO in Israel. These two limitations may cause a selection bias; PCPs who use more mHealth apps or are more technologically inclined in general were more likely to respond to this questionnaire, and therefore, the results may not represent the entire population of PCPs in Israel. Another limitation is the use of a questionnaire that has not been validated.

The strengths of this study are its nationwide coverage of PCPs from all areas of Israel and the representation of PCPs from all age groups.

### Comparison with prior work

In this study, we report a relatively high rate of PCPs who use mHealth apps (77.9%). This finding is in line with studies conducted in Austria [27], eastern China, Turkey, and Australia, where most PCPs reported using at least one mHealth app [32, 33]. However, in a study conducted among French PCPs, only 19.5% reported using connected health devices [36]. We found that relatively younger physicians were more likely to use mHealth apps. This finding is in line with those of other studies [27]. All these studies were done using a questionnaire, but due to different models of primary care in different countries, are not easily comparable.

The use of telemedicine apps by PCPs in Israel includes the HMO app, through which communication with patients is possible, including sending chronic medication and sick leaves and answering questions sent by the patients. WhatsApp is usually used for communication and consultation with other PCPs or with specialists from other fields of medicine.

The least frequently used apps were medical calculators and differential diagnosis applications, although these apps are considered accurate and reliable [37]. This is an important aspect since many try to develop these tools in the tech world [38].

The reasons for the use of mHealth apps in daily routine have been examined by several studies and reviews. The main factors found to influence physicians are usefulness, ease of use, design, cost, time considerations, compatibility, content, privacy and security issues, personalization, and interactions with peers. Social and organizational factors, including workflow, have also been found to affect physicians, as have patient-related factors, policy and regulations, physicians’ attitudes and social influence, monetary factors, evidence-based data, and user engagement [6, 22].

The possibilities for managing patients with chronic conditions with the assistance of mHealth apps are promising [21]. Apps can assist in the management of asthma, chronic pulmonary disease, heart failure, diabetes mellitus, and hypertension and can contribute to improved compliance both in the treatment of infectious diseases (tuberculosis, HIV) and in weight control [39, 40]. Nonetheless, physicians are hesitant to prescribe mHealth apps to their patients; only 6% of the PCPs in our study did so. This phenomenon has also been noted in other studies. In Germany, for example, certain mHealth apps were approved by the state, yet while 62% of physicians reported that they supported prescribing these apps, only 30% reported that they planned to do so [30]. Barriers to prescribing mHealth apps to patients included lack of information, reimbursement issues and legal concerns, medical evidence (or lack thereof), and technological difficulties. In Sweden, only a small group of physicians recommends the use of mHealth apps to their patients, mainly due to lack of evidence-based data and multi-language support [31].

Sarradon-Eck and colleagues report on factors that encourage PCPs to prescribe mHealth apps to patients [34]. They recognize that while some PCPs are in favor of prescribing these apps, others are concerned about risks for patients, data privacy and security, overmedicalization, and the possibility of increasing healthcare inequalities). Other concerns were related to physicians themselves and included fear of additional tasks, depersonalization of the patient–doctor interaction, and worries regarding increased drug prescriptions. Almost two-thirds of PCPs in a study conducted in Belgium indicated that they were not interested in prescribing mHealth apps to their patients. Problems with data exchangeability and time constraints were reported as the main reasons for this attitude [35]. Interestingly, in a study conducted in Australia, over half of PCPs recommended mHealth apps to patients [29]. The barriers reported by them included a lack of knowledge regarding the effectiveness of these apps and concerns regarding inaccurate or biased sources of information.

## Conclusions

mHealth apps are widely used by PCPs, regardless of physician gender or seniority. Information from mHealth apps is considered reliable by PCPs. The main barriers to app use are unfamiliarity with relevant applications and a preference for computers over smartphones. Nevertheless, PCPs do not advise their patients to use mHealth apps, and the reasons for this should be further evaluated.

### Electronic supplementary material

Below is the link to the electronic supplementary material.

Supplementary Material 1

Supplementary Material 2

Supplementary Material 3

## Data Availability

The data underlying this article will be shared on reasonable request to the corresponding author.
