# Supplementary material for: The use of medical health applications by primary care physicians in Israel: a cross-sectional study
Source: BMC Health Serv Res. 2024 Apr 2;24:410. doi: 10.1186/s12913-024-10880-w (PMC10988819; doi:10.1186/s12913-024-10880-w)
Supplement: Supplementary file 1 — Supplementary Material 1 [file 12913_2024_10880_MOESM1_ESM.docx]

| **Responses to the questionnaire** | | |
| --- | --- | --- |
|  |  |  |
| **Question** | **N** | **%** |
| 1. **Do you possess a smartphone?** |  |  |
| Yes | 181 | 94.8 |
| No (including possession of special smartphones used for messages and calls only) | 10 | 5.2 |
|  |  |  |
| 1. **During the past week, how often did you use smartphone applications for personal use (such as Waze or WhatsApp)?** |  |  |
| Not at all | 1 | 6.0 |
| Once | 4 | 2.2 |
| Several times | 10 | 5.5 |
| Daily | 32 | 17.7 |
| Multiple times daily | 134 | 74.0 |
|  |  |  |
| 1. **Do you tend to use medical applications (such as Micromedex, MedCalc, etc.)?** |  |  |
| Yes | 123 | 68 |
| No | 58 | 32 |
|  |  |  |
| 1. **During the past week, how often did you use medical applications in the course of your work?** |  |  |
| Not at all | 40 | 22.1 |
| Once | 33 | 18.2 |
| Several times | 62 | 34.3 |
| Daily | 25 | 13.8 |
| Multiple times daily | 21 | 11.6 |
|  |  |  |
| 1. **How many medical applications are currently on your smartphone home screen?** |  |  |
| 0 | 46 | 25.4 |
| 1 | 23 | 12.7 |
| 2 | 34 | 18.8 |
| 3 or more | 72 | 39.8 |
| *No response | 6 | 3.3 |
| 1. **During the past week, how often did you use medical applications in the course of your work for information on medications or dosage?** |  |  |
| Not at all | 58 | 32.0 |
| Once | 34 | 18.8 |
| Several times | 51 | 28.2 |
| Daily | 6 | 3.3 |
| Multiple times daily | 16 | 8.8 |
| *No response | 16 | 8.8 |
| 1. **During the past week, how often did you use medical applications in the course of your work for medical calculations (such as calculating renal clearance or various medical scores)?** |  |  |
| Not at all | 93 | 51.4 |
| Once | 38 | 21.0 |
| Several times | 21 | 11.6 |
| Daily | 9 | 5.0 |
| Multiple times daily | 3 | 1.7 |
| *No response | 17 | 9.4 |
| 1. **During the past week, how often did you use medical applications in the course of your work to access scientific databases or articles?** |  |  |
| Not at all | 43 | 23.8 |
| Once | 32 | 17.7 |
| Several times | 54 | 29.8 |
| Daily | 18 | 9.9 |
| Multiple times daily | 17 | 9.4 |
| *No response | 17 | 9.4 |
| 1. **During the past week, how often did you use medical applications in the course of your work for differential diagnoses?** |  |  |
| Not at all | 93 | 51.4 |
| Once | 31 | 17.1 |
| Several times | 27 | 14.9 |
| Daily | 7 | 3.9 |
| Multiple times daily | 5 | 2.8 |
| *No response | 18 | 9.9 |
| 1. **During the past week, how often did you use medical applications in the course of your work to select a treatment?** |  |  |
| Not at all | 81 | 44.8 |
| Once | 43 | 23.8 |
| Several times | 27 | 14.9 |
| Daily | 6 | 3.3 |
| Multiple times daily | 7 | 3.9 |
| *No response | 17 | 9.4 |
| 1. **During the past week, how often did you use medical applications in the course of your work for teleconsultations?** |  |  |
| Not at all | 49 | 27.1 |
| Once | 24 | 13.3 |
| Several times | 41 | 22.7 |
| Daily | 5 | 2.8 |
| Multiple times daily | 41 | 22.7 |
| *No response | 21 | 11.6 |
| 1. **During the past week, did you use medical applications for other reasons? (reason specified in free text)** |  |  |
| WhatsApp | 4 | 2.2 |
| Maccabi App | 1 | 0.6 |
| UpToDate | 7 | 3.9 |
| Google | 1 | 0.6 |
| 1. **Why do you use medical applications?** |  |  |
| Time efficiency | 92 | 50.8 |
| Accessibility | 97 | 53.6 |
| Up-to-date and reliable information | 92 | 50.8 |
| 1. **How many medical applications do use daily?** |  |  |
| 0 | 34 | 18.8 |
| 1 | 56 | 30.9 |
| 2 | 32 | 17.7 |
| 3 or more | 36 | 19.9 |
| *No response | 23 | 12.7 |
| 1. **What would encourage you to use medical applications more frequently**? |  |  |
| Colleague recommendation | 57 | 31.5 |
| Regulatory approval | 49 | 27.1 |
| Assurance of information credibility | 76 | 42.0 |
| Statement regarding conflict of interest from application developers | 45 | 24.9 |
| Free or subsidized use | 88 | 48.6 |
| Formal guidance on application use | 40 | 22.1 |
| Knowledge of time saved | 87 | 48.1 |
| Monetary benefit | 38 | 21.0 |
| 1. **What are the reasons you use medical applications infrequently?** (presented only to infrequent users according to the answer to Question 4) |  |  |
| Unfamiliar with relevant applications | 22 | 30.1 |
| More comfortable using a computer | 33 | 45.2 |
| Not comfortable using the phone in patients’ presence | 9 | 12.3 |
| Concerns regarding information reliability | 4 | 5.5 |
| Concerns regarding patient privacy or other ethical considerations | 3 | 4.1 |
| Prefer to rely on my own knowledge | 5 | 6.8 |
| Prefer to rely on published articles and texts | 5 | 6.8 |
| 1. **Do you recommend medical applications to patients?** |  |  |
| Never | 70 | 38.7 |
| Infrequently | 37 | 20.4 |
| Sometimes | 29 | 16.0 |
| Often | 4 | 2.2 |
| Frequently | 7 | 3.9 |
| *No response | 34 | 18.8 |
| 1. **Would you participate in training that includes recommendations regarding the use of medical applications?** |  |  |
| No | 14 | 7.7 |
| Probably not | 30 | `16.6 |
| Probably | 59 | 32.6 |
| Definitely | 38 | 21.0 |
| Don’t know | 6 | 3.3 |
| *No response | 34 | 18.8 |
| 1. **In the future, how frequently do you think you will use medical applications?** |  |  |
| Less frequently | 3 | 1.7 |
| The same | 41 | 22.7 |
| More frequently | 103 | 56.9 |
| *No response | 34 | 18.8 |
