# Supplementary material for: The use of medical health applications by primary care physicians in Israel: a cross-sectional study
Source: BMC Health Serv Res. 2024 Apr 2;24:410. doi: 10.1186/s12913-024-10880-w (PMC10988819; doi:10.1186/s12913-024-10880-w)
Supplement: Supplementary file 2 — Supplementary Material 2 [file 12913_2024_10880_MOESM2_ESM.docx]

|  |
| --- |
| Dear physician,  We are conducting a study about patterns of use of medical applications among primary care physicians in Israel. All collected data is confidential and will only be used for research purposes.  This study is neither funded nor supported by any commercial element, and the researchers have no interest in conducting this study beyond its academic and research purpose.  You may choose to answer all or some of the questions in this questionnaire, and you may stop at any point. Your identity will remain confidential.  Thank you for your cooperation. |
| 1. **Do you possess a smartphone? (multiple choice)** |
| Yes |
| No (including possession of special smartphones used for messages and calls only) |
|  |
| 1. **During the past week, how often did you use smartphone applications for personal use (such as Waze or WhatsApp)? (multiple choice)** |
| Not at all |
| Once |
| Several times |
| Daily |
| Multiple times daily |
|  |
| 1. **Do you use medical applications (such as Micromedex, MedCalc, etc.)? (multiple choice)** |
| Yes |
| No |
|  |
| 1. **During the past week, how often did you use medical applications in your work? (multiple choice)** |
| Not at all |
| Once |
| Several times |
| Daily |
| Multiple times daily |
|  |
| 1. **How many medical applications are currently on your smartphone home screen? (multiple choice)** |
| 0 |
| 1 |
| 2 |
| 3 or more |
| *No response |
| 1. **During the past week, how often did you use medical applications in your work for information on medications or dosages? (multiple choice)** |
| Not at all |
| Once |
| Several times |
| Daily |
| Multiple times daily |
| *No response |
| 1. **During the past week, how often did you use medical applications your work for medical calculations (such as calculating renal clearance or various medical scores)? (multiple choice)** |
| Not at all |
| Once |
| Several times |
| Daily |
| Multiple times daily |
| *No response |
| 1. **During the past week, how often did you use medical applications in your work to access scientific databases or articles? (multiple choice)** |
| Not at all |
| Once |
| Several times |
| Daily |
| Multiple times daily |
| *No response |
| 1. **During the past week, how often did you use medical applications in your work for differential diagnoses? (multiple choice)** |
| Not at all |
| Once |
| Several times |
| Daily |
| Multiple times daily |
| *No response |
| 1. **During the past week, how often did you use medical applications in your work to select an appropriate treatment? (multiple choice)** |
| Not at all |
| Once |
| Several times |
| Daily |
| Multiple times daily |
| *No response |
| 1. **During the past week, how often did you use medical applications in your work for telemedicine activities? (multiple choice)** |
| Not at all |
| Once |
| Several times |
| Daily |
| Multiple times daily |
| *No response |
| 1. **During the past week, did you use medical applications for other reasons? (free text)** |
| 1. **Why do you use medical applications? (multiple choice; choose one or more; free text)** |
| Time efficiency |
| Accessibility |
| Up-to-date and reliable information  Other: ______________ |
| 1. **How many medical applications do use daily? (multiple choice)** |
| 0 |
| 1 |
| 2 |
| 3 or more |
| *No response |
| 1. **What would encourage you to use medical applications more frequently**? **(multiple choice; choose one or more; free text)** |
| Colleague recommendation |
| Regulatory approval |
| Assurance of information credibility |
| Statement regarding conflict of interest from application developers |
| Free or subsidized use |
| Formal guidance on application use |
| Knowledge of time saved |
| Monetary benefit  Other:__________ |
|  |
| 1. **What are the reasons you use medical applications infrequently?** (presented only to infrequent users according to the answer to Question 4) **(multiple choice; choose one or more; free text)** |
| Unfamiliar with relevant applications |
| More comfortable using a computer |
| Not comfortable using the phone in patients’ presence |
| Concerns regarding information reliability |
| Concerns regarding patient privacy or other ethical considerations |
| Prefer to rely on my own knowledge |
| Prefer to rely on published articles and texts  Other:__________ |
| 1. **Do you recommend medical applications to patients for monitoring and treatment? (multiple choice)** |
| Never |
| Infrequently |
| Sometimes |
| Often |
| Frequently |
| *No response |
| 1. **Would you participate in training that includes recommendations regarding the use of medical applications? (multiple choice)** |
| No |
| Probably not |
| Probably |
| Definitely |
| Don’t know |
| *No response |
| 1. **In the future, how frequently do you think you will use medical applications? (multiple choice)** |
| Less frequently |
| The same |
| More frequently |
| *No response  #If you expect to use medical applications less frequently, please elaborate as to the reason: ___________ **(free text)**  #If you expect to use medical applications more frequently, please elaborate as to the reason: ______ **(free text)**  And for what needs **(multiple choice; choose one or more; free text)**:  Information about medications and dosages  Medical calculators  Access to information and articles  Diagnostic assistance  Treatment selection  Telemedicine  Other: ____  **Please answer the following demographic questions**  **20. Gender (multiple choice)**  Male  Female  **21. Year of birth (free text)**  ____________  **22. Region of residence (multiple choice):**  South  Central  Sharon  Jerusalem  North  **23. Country of birth (multiple choice):**  Israel  Eastern Europe and former USSR  Western Europe and USA  South America  Other  **25. Country of medical school attendance (multiple choice):**  Israel  Eastern Europe and former USSR  Western Europe and USA  South America  Other  **26. Year of medical school graduation (free text):**  **______________**  **27. How would you characterize your primary clinic (multiple choice; free text):**  An urban clinic  A rural clinic  A hospital  Other: _______________  **28. Region of practice (multiple choice):**  North  South  Central  Jerusalem  **29. Employment status (multiple choice):**  Hired  Independent  Both  **30. Specialty (multiple choice):**  GP  Family medicine resident  Family medicine specialist  Internal medicine specialist  Other  **Thank you for your cooperation!** |
